# A phase 1 dose-escalation study of veliparib with bimonthly FOLFIRI in patients with advanced solid tumours

**DOI:** 10.1038/s41416-018-0003-3

**Published:** 2018-03-12

**Authors:** Jordan Berlin, Ramesh K. Ramanathan, John H. Strickler, Deepa S. Subramaniam, John Marshall, Yoon-Koo Kang, Robert Hetman, Matthew W. Dudley, Jiewei Zeng, Caroline Nickner, Hao Xiong, Philip Komarnitsky, Stacie Peacock Shepherd, Herbert Hurwitz, Heinz-Josef Lenz

**Affiliations:** 1 Vanderbilt-Ingram Cancer Center, Nashville, TN USA; 2 Translational Genomics Research Institute – Virginia G. Piper Cancer Center, Scottsdale, AZ USA; 3 Duke University Medical Center, Durham, NC USA; 4 Lombardi Comprehensive Cancer Center, Georgetown University, Washington, DC USA; 5 Asan Medical Center, University of Ulsan College of Medicine, Seoul, South Korea; 6 AbbVie Inc, North Chicago, IL USA; 7 Division of Medical Oncology, Keck School of Medicine, University of Southern California, Los Angeles, CA USA; 8 Present Address: Corcept Therapeutics, Menlo Park, CA USA

**Keywords:** Drug development, Phase I trials

## Abstract

**Background:**

Veliparib is a potent poly(ADP-ribose) polymerase inhibitor. This phase 1 study aimed to establish the maximum tolerated dose (MTD) and recommended phase 2 dose (RP2D) of veliparib combined with various FOLFIRI regimens in patients with solid tumours.

**Methods:**

Patients received veliparib (10–270 mg BID, days 1–5, 15–19) and FOLFIRI (days 1–3, 15–17) in three regimens containing 5-fluorouracil 2,400 mg/m^2^: irinotecan 150 mg/m^2^ and folinic acid 400 mg/m^2^ (part 1); irinotecan 180 mg/m^2^, folinic acid 400 mg/m^2^, and 5-fluorouracil 400 mg/m^2^ bolus (part 2), or irinotecan 180 mg/m^2^ (part 3). The RP2D was further evaluated in safety expansion cohorts. Preliminary antitumour activity was also assessed.

**Results:**

Ninety-two patients received ≥1 veliparib dose. MTD was not reached; RP2D was set at 200 mg BID veliparib plus FOLFIRI (without 5-fluorouracil bolus). Most common treatment-emergent adverse events were neutropenia (66.3%), diarrhoea, and nausea (60.9% each). Dose-limiting toxicities (*n* = 4) were grade 3 gastritis and grade 4 neutropenia and febrile neutropenia. Veliparib exposure was dose-proportional, with no effects on the pharmacokinetics of FOLFIRI components. Fifteen patients had a partial response (objective response rate, 17.6%).

**Conclusions:**

The acceptable safety profile and preliminary antitumour activity of veliparib plus FOLFIRI support further evaluation of this combination.

## Introduction

Poly(ADP-ribose) polymerase-1 (PARP-1) and PARP-2 are abundant nuclear enzymes that recognise DNA damage, enable DNA repair, and prevent cellular cytotoxicity.^1,2^ PARPs play a vital role in maintaining genomic stability as part of normal cellular physiology. Notably, these enzymes are overexpressed in several cancer types and are implicated in tumourigenesis. Thus, PARP inhibition is a valid target for anticancer therapy.^3,4^

Genomic instability with increased response to DNA damage is a common feature of cancer. For many patients with cancer, DNA-damaging agents, including cytotoxic chemotherapy and radiation therapy, remain a mainstay of treatment. Genetic deficiencies, together with the action of DNA-damaging chemotherapies, render cancer cells more dependent on PARP-1 and PARP-2 for DNA repair and therefore more sensitive to PARP inhibition. Such an antitumour strategy is neither inherently cytotoxic nor mutagenic.^5^ PARP inhibition leads to the accumulation of DNA single-strand and double-strand breaks at replication forks. These DNA strand breaks are normally repaired by the homologous recombination repair (HRR) pathway, for which key components include the tumour suppressor proteins BRCA1 and BRCA2.^6^ Therefore, PARP inhibition has potential as targeted therapy for cancers with underlying defects in HRR, such as *BRCA*-mutated tumours and platinum-sensitive ovarian cancers.^6,7^ PARP inhibition may also serve as a sensitiser, potentiating the activity of a variety of DNA-damaging agents, including topoisomerase inhibitors, alkylators, and platinum-based agents.^8–10^

In preclinical models, synergy has been demonstrated between PARP inhibitors and topoisomerase I (Top 1) inhibitors, such as irinotecan.^11,12^ Top 1 was shown to be an acceptor of PAR polymers, with PARP-1 catalyzing the synthesis and attachment of highly negatively charged PARs to target proteins, such as Top 1.^13^ PARP inhibitors, like veliparib, abrogate Top 1-PARylation and Top 1 efflux from the nucleolus to the nucleoplasm. This process further facilitates enhanced trapping of Top 1 in the nucleus with Top 1 poisons.^14^ Treatment with the Top 1 inhibitor irinotecan can cause formation of a cleavable complex that involves Top 1 covalently attached to the DNA 3′ phosphate. Top 1-associated single-strand breaks activate PARP-1. Through the recruitment of X-ray repair cross-complementing protein 1 (XRCC1), these breaks also promote removal of the cleavage complex by tyrosyl DNA phosphodiesterase-1 (TDP1) and subsequent completion of DNA repair.^15–17^ Therefore, inhibition of PARP potentiates irinotecan-induced DNA damage by disabling the PARP1-XRCC1-TDP1 repair pathway.^18,19^

5-fluorouracil (5-FU) is a pyrimidine analog and antimetabolite with the ability to incorporate into the DNA molecule and stop synthesis. The mechanism of 5-FU cytotoxicity is based on misincorporation of fluoronucleotides into the DNA strand, followed by inhibition of the nucleotide synthetic enzyme thymidylate synthase. This in turn blocks the reductive methylation of deoxyuridine monophosphate to deoxythymine monophosphate (dTMP), thus inhibiting dTMP synthesis^20,21^ and the process of DNA synthesis/repair. Combining the use of PARP inhibitors with antitumour drugs that display similar mechanisms of action, such as irinotecan and 5-FU, has the potential to enhance outcomes of patients with cancers receiving various chemotherapeutic regimens. Additionally, based on the mechanisms described above, treatment regimens containing 5-FU, irinotecan, and folinic acid (FOLFIRI) are widely used in first- and second-line metastatic colorectal cancer.^22,23^

Veliparib (ABT-888) is a potent, oral PARP-1 and PARP-2 inhibitor shown to enhance the antitumour activity of chemotherapy and radiation therapy in various preclinical tumour models.^24,25^ In colon cancer cell lines, veliparib synergise with irinotecan to induce cell death,^11^ and a similar effect has been reported with veliparib and radiation combined with 5-FU, oxaliplatin, and irinotecan.^26^ Preliminary evidence of antitumour activity of veliparib was also noted in the clinical setting in rectal cancer.^10^ The present study describes the safety, pharmacokinetic (PK) profile, and preliminary efficacy results of veliparib in combination with FOLFIRI in patients with advanced solid tumours. Because the goal of this study was to inhibit PARP to improve chemotherapy outcomes, veliparib was administered in an intermittent manner only around the dosing of FOLFIRI.

## Patients and Methods

### Study design

**Fig. 1:**
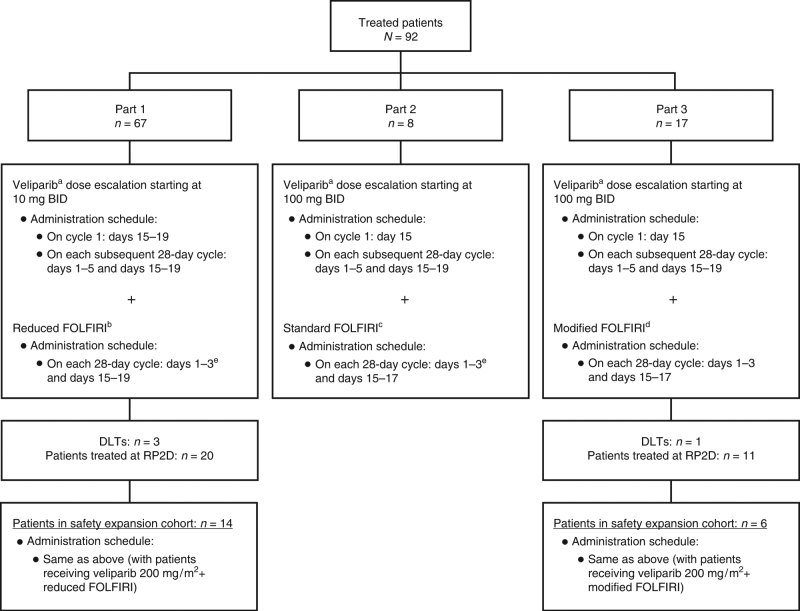
Study design. **a** Veliparib was administered in all three study parts 1 h prior to FOLFIRI infusion. **b** Reduced FOLFIRI = reduced dose of irinotecan 150 mg/m^2^ (90-min infusion) + folinic acid 400 mg/m^2^ (2-h infusion during irinotecan administration) + 5-FU 2,400 mg/m^2^ (46-h continuous infusion immediately following irinotecan administration). **c** Standard FOLFIRI = standard dose of irinotecan 180 mg/m^2^ (90-min infusion) + folinic acid 400 mg/m^2^ (2-h infusion during irinotecan administration) + 5-FU 400 mg/m^2^ (bolus immediately following irinotecan administration) and 2,400 mg/m^2^ (46-h continuous infusion). **d** Modified FOLFIRI = modified dose of irinotecan 180 mg/m^2^ (90-min infusion) + 5-FU 2,400 mg/m^2^ (46-h continuous infusion immediately following irinotecan administration). **e** The 400 mg/m^2^ bolus 5-FU dose was not tolerated during the first 2 weeks of cycle 1, before veliparib administration. Therefore, in part 1 of the study, only 4/67 patients received 400 mg/m^2^ of 5-FU bolus infusions starting cycle 2. For the remaining 63 patients, bolus administration of 5-FU was removed to reduce the toxic effects of 5-FU. Part 2 dose escalation was consequently discontinued and, although patients could continue veliparib, they were considered not evaluable. Data from part 2 were combined with those from part 3. 5-FU 5-fluorouracil, BID twice daily, DLT dose-limiting toxicity, FOLFIRI 5-fluorouracil plus folinic acid plus irinotecan, RP2D recommended phase 2 dose

This phase 1 open-label, multicentre, dose-escalation, and safety expansion study evaluated veliparib in combination with bimonthly FOLFIRI in patients with advanced solid tumours. The primary objectives were to determine the maximum tolerated dose (MTD) and establish the recommended phase 2 dose (RP2D) of the combination therapy. Secondary objectives were to assess the safety and tolerability, PK profile, and exploratory efficacy of the combination for each of the FOLFIRI regimens. Patients received veliparib and bimonthly FOLFIRI in 28-day cycles. Veliparib was administered on days 15–19 in cycle 1 and on days 1–5 and 15–19 in subsequent cycles (Fig. 1). Patients with stable disease or better status could continue veliparib and bimonthly FOLFIRI at the investigator’s discretion until progressive disease (PD), unacceptable toxicity, or FOLFIRI discontinuation. The trial was registered with the ClinicalTrials.gov registry (NCT01123876) and was approved by appropriate independent ethics committees/institutional review boards prior to initiation. The study was performed in accordance with the 1964 Declaration of Helsinki and its later amendments. Written informed consent was obtained from all patients before study enrollment.

### Patients

Eligible patients (≥18 years of age) had histologically or cytologically confirmed solid tumours that were metastatic or unresectable, for which FOLFIRI was a viable therapeutic option or no standard curative therapeutic options were available. Alternatively, the patient had to have disease refractory to standard therapy or histologically confirmed gastric cancer (patients from South Korea only). In addition, patients were required to have an Eastern Cooperative Oncology Group (ECOG) performance status 0–1, and to have received prior therapy with ≤3 DNA-damaging agents or cytotoxic chemotherapies within the past 2 years. Patients were also required to have adequate haematologic function (absolute neutrophil count ≥ 1500 per mm^3^, platelets ≥ 100,000 per mm^3^, haemoglobin ≥ 9.5 g/dL), renal function (serum creatinine ≤ 1.5× upper limit of normal (ULN) or creatinine clearance ≥ 50 mL/min per 1.73 m^2^), and hepatic function (bilirubin ≤ 1.5× ULN, and aspartate aminotransferase and alanine aminotransferase ≤ 2.5× ULN, or ≤5× ULN for patients with liver metastases). Exclusion criteria were: prior anticancer therapy (including chemotherapy, immunotherapy, radiotherapy, biologic therapy, or any investigational therapy) within 28 days prior to study drug administration; known history of brain metastases and primary central nervous system tumours; and previous exposure to irinotecan, or known hypersensitivity to irinotecan, 5-FU, or folinic acid.

### Treatment schedule

Dose escalation occurred in three parts to evaluate the MTD/RP2D of three different bimonthly FOLFIRI regimens in combination with veliparib (Fig. **1**). A minimum of three patients per dose level were included for dose-toxicity modeling. In part 1, dose escalation began with veliparib twice daily (BID) on days 15–19 of cycle 1 (to allow the determination of single-agent PK for irinotecan, the active metabolite of irinotecan (SN-38), folinic acid, and 5-FU on cycle 1 day 1). In parts 2 and 3, dose escalation began with veliparib BID on day 15 of cycle 1. The differences in the dosing schedules of parts 1, 2, and 3 relate to dosing of irinotecan and 5-FU, and are detailed in Fig. 1. In all parts, veliparib continued to be administered on days 1–5 and 15–19 of each subsequent 28-day cycle. Veliparib was administered 1 h prior to FOLFIRI infusion (Supplementary Table S1). Enrollment into parts 2 and 3 occurred concurrently with enrollment into part 1, once dose level in part 1 exceeded the threshold of veliparib 150-mg BID. Dose escalation was guided by a Bayesian continual reassessment method (CRM) that incorporated information from all prior events, such as doses and tolerability, into a statistical dose-response model. This model guided the selection of subsequent doses in real time as the trial progressed. The statistical analysis was based on a logistic regression model for the dose-toxicity relationship (i.e., the relationship between dose and the probability of dose-limiting toxicities (DLTs)). Maximum tolerated dose was defined as the highest dose at which <33% of patients experienced a DLT during the second half of cycle 1. The RP2D was defined by observed DLTs and determinations of MTD. The RP2D was further evaluated in two safety expansion cohorts that included patients for whom any irinotecan-based regimen would be considered standard therapy, with ~50% of patients having a diagnosis of gastric cancer.

Prophylactic colony-stimulating factors were not allowed during cycle 1 for the dose-escalation cohorts and the expanded safety cohorts. For subsequent cycles, prophylactic and treatment usage of colony-stimulating factors was allowed per investigator’s standard practice and/or American Society of Clinical Oncology guidelines.

### Safety, pharmacokinetic, and efficacy assessments

Treatment-emergent adverse events (TEAEs) were assessed from the time of study drug administration until 30 days following discontinuation of study drug. TEAEs were assessed according to the National Cancer Institute Common Terminology Criteria for Adverse Events version 4.0. In dose-escalation cohorts, blood samples were collected on day 15 of cycle 1 for veliparib PK, and on days 1 and 15 of cycle 1 for PK assessment of the FOLFIRI components irinotecan, SN-38, folinic acid (leucovorin), and 5-FU. Exploratory efficacy end points included objective response rate (ORR), time to disease progression (TTP), duration of overall response (DOR), and ECOG performance status. Objective response rate included confirmed complete response (CR) and partial response (PR). Objective response rate evaluation was based on Response Evaluation Criteria in Solid Tumours version 1.1. Objective response rate was calculated for all patients with one or more measurable lesions at baseline. Time to disease progression was defined as the number of days from the date the patient started study drug to the date of the patient’s disease progression. Duration of overall response was defined as the number of days from the day criteria were met for CR or PR (whichever was recorded first) to the date that PD was objectively documented. If a patient was still responding, the patient’s data were censored at the date of the last study visit at which a tumour assessment was performed. Analyses of change and/or percentage change from baseline for tumour size were performed for each scheduled post-baseline visit and for the final visit.  ECOG performance status was assessed at all visits, with descriptive statistics summarised for each assessment. In addition, a mean change from baseline to each assessment time was summarised.

### Statistical analyses

The sample size was based on clinical justification. No specific statistical hypothesis tests were planned, with descriptive statistics used for the analysis of safety, PK, and tumour response data. The study population for all safety and preliminary efficacy analyses included patients who received one or more doses of study medication. Data from parts 1–3 of the dose-escalation cohort were combined for assessment of the effect of veliparib on the PK of irinotecan, SN-38, and folinic acid. Linear mixed effects models were performed on natural logarithmically transformed maximum plasma concentration (*C*_max_) and area under the plasma or serum concentration-time curve (AUC) to compare exposure of irinotecan, SN-38, and folinic acid with and without the administration of veliparib. Point estimates and the corresponding 90% confidence intervals (CIs) of the ratio of central values with and without veliparib administration were calculated.

## Results

### Patient demographics and baseline characteristics

Ninety-seven patients were enrolled and the study was performed between 25 February 2010 and 7 January 2015. Of these, 92 patients received one or more doses of veliparib. Of the treated patients, 67 received veliparib plus FOLFIRI containing irinotecan 150 mg/m^2^ (part 1), and 25 patients received veliparib plus FOLFIRI containing irinotecan 180 mg/m^2^ (parts 2 and 3). All 92 patients were discontinued from the study; 77 (83.7%) patients discontinued primarily due to PD. Demographics and baseline characteristics are summarised in Table 1. Briefly, the majority of patients were male (54.3%) and had a median age of 56.5 years (range: 24–77). The median number of prior oncology regimens was two (range, 0–9). Nineteen patients (20.7%) had received prior oxaliplatin treatment; the same number had received prior treatment with paclitaxel. Patients were most commonly diagnosed with gastric (23.5%) or pancreatic (16.5%) cancer, and had received no previous treatment with a PARP inhibitor. There were no clinically meaningful differences between patients in part 1, and parts 2 and 3 in terms of demographics and baseline characteristics.

**Table 1:** Demographic and baseline characteristics (safety analysis set)

Characteristics	Part 1^a^ *n* = 67	Parts 2 and 3^b^ *n* = 25	Total *n* = 92
Female, *n* (%)	31 (46.3)	11 (44.0)	42 (45.7)
Male, *n* (%)	36 (53.7)	14 (56.0)	50 (54.3)
*Age, years*			
Median (range)	58.0 (24–77)	46.0 (30–73)	56.5 (24–77)
<65, *n* (%)	53 (79.1)	22 (88.0)	75 (81.5)
≥65, *n* (%)	14 (20.9)	3 (12.0)	17 (18.5)
*Race*			
White	49 (73.1)	19 (76.0)	68 (73.9)
African American	3 (4.5)	5 (20.0)	8 (8.7)
Asian	13 (19.4)	0 (0)	13 (14.1)
Other	2 (3.0)	1 (4.0)	3 (3.3)
Weight, kg, median (range)	72.0 (47.0–119.0)	81.0 (56.0–121.0)	73.0 (47.0–121.0)
BMI, kg/m^2^, median (range)	25.7 (17.1–48.7)	27.3 (22.5–42.2)	26.2 (17.1–48.7)
*ECOG performance status, n (%)*			
0			45 (48.9)
1	—	—	47 (51.1)
*Prior oncology medications, n (%)*			
0	—	—	2 (2.2)
1	—	—	16 (17.4)
2	—	—	27 (29.3)
≥3	—	—	47 (51.1)
Gemcitabine	—	—	21 (22.8)
Oxaliplatin	—	—	19 (20.7)
Paclitaxel	—	—	19 (20.7)
*Tumour type, n (%)*			
Breast	—	—	9 (10.6)
Colorectal	—	—	10 (10.9)
Gastric	—	—	20 (23.5)
Ovarian	—	—	9 (10.6)
Pancreatic	—	—	14 (16.5)
Other	—	—	30 (32.6)

*BMI* body mass index, *ECOG* Eastern Cooperative Oncology Group

^a^Irinotecan 150 mg/m^2^

^b^Irinotecan 180 mg/m^2^

^c^All oncology medications started on the same day were considered as components of a single oncology therapy regimen and counted accordingly

### Safety and tolerability

The median number of treatment cycles among all treated patients was four (range: 1–54 cycles). On the basis of the Bayesian CRM guiding dose escalation, the MTD was not reached; veliparib dose was not escalated beyond 270 mg BID. DLTs occurred in four patients: in part 1, two DLTs of grade 4 neutropenia (160 mg and 270 mg veliparib BID) and one DLT of grade 3 severe gastritis (270 mg veliparib BID) were reported; in part 3, one DLT of grade 4 febrile neutropenia occurred (100 mg veliparib BID). In part 2, the 5-FU bolus reduced the tolerability of the FOLFIRI regimen. This effect was also observed during the first 2 weeks of cycle 1 prior to veliparib administration. As a result, the part 2 dose-escalation was discontinued, the bolus was excluded, and a RP2D with bolus 5-FU as part of the FOLFIRI regimen was not determined. Based on the two DLTs observed at 270 mg BID in part 1, and the increased nausea at doses > 200 mg BID, the RP2D of veliparib was established at 200 mg BID with bimonthly FOLFIRI (irinotecan 150 mg/m^2^ or 180 mg/m^2^ without 5-FU bolus). In two safety expansion cohorts, patients were enrolled at the veliparib RP2D plus FOLFIRI, containing either irinotecan 150 mg/m^2^ (gastric cancer, *n* = 14) or 180 mg/m^2^ (colorectal cancer, *n* = 6).

All treated patients experienced one or more TEAEs. The most common TEAEs (reported in ≥30% of patients) included: neutropenia (66.3%), diarrhoea (60.9%), nausea (60.9%), vomiting (47.8%), fatigue (47.8%), anaemia (44.6%), and alopecia (43.5%). Treatment-emergent adverse events are summarised in Table 2. The type and incidence of TEAEs were similar for patients who received FOLFIRI containing 150 mg/m^2^ or 180 mg/m^2^ irinotecan, and were consistent with the known side effects of FOLFIRI. Sixty-two (67.4%) patients experienced ≥1 grade 3 or 4 TEAEs. The most common grade 3 or 4 TEAEs were neutropenia (42.4%) and anaemia (9.8%). Thirty-five (38.0%) patients experienced ≥1 treatment-emergent serious AEs (SAEs). Serious AEs that occurred in ≥2 patients were: pyrexia (4.3%), vomiting (3.3%), and febrile neutropenia, abdominal pain, intestinal obstruction, nausea, PD, dehydration, and malignant neoplasm progression (2.2% each). The median duration of veliparib treatment was 33.0 days (range: 4–449 days). Five (5.4%) patients discontinued veliparib due to TEAEs of hypokalemia, blood alkaline phosphatase increase, clavicle fracture, malignant neoplasm progression, and neutropenia. Neutropenia was the only event that was considered by the investigator to be at least possibly related to veliparib. Few patients had grade ≥ 3 haematologic or chemistry laboratory abnormalities recorded as SAEs or TEAEs that led to veliparib discontinuation. Four patients had TEAEs that led to death within 30 days of the last veliparib dose (*n* = 4; cardiac arrest and disease progression, hydrocephalus, malignant pleural effusion, and malignant neoplasm progression). One patient had a TEAE that led to death within 63 days after the last veliparib dose (*n* = 1; disease progression). All TEAEs leading to death were considered by the investigator as not related or probably not related to veliparib and FOLFIRI.

**Table 2:** Treatment-emergent adverse events occurring in ≥30% of all patients (safety analysis set)

	Part 1 *n* = 67		Parts 2^a^ and 3 *n* = 25		Combined RP2D^b^ *n* = 31		Total *n* = 92	
Adverse event, *n* (%)	All grades	Grade 3/4	All grades	Grade 3/4	All grades	Grade 3/4	All grades	Grade 3/4
Neutropenia	41 (61.1)	26 (38.8)	20 (80.0)	13 (52.0)	25 (80.6)	17 (54.8)	61 (66.3)	39 (42.4)
Diarrhoea	40 (59.7)	5 (7.4)	16 (64.0)	0 (0.0)	17 (54.8)	5 (16.1)	56 (60.9)	5 (5.4)
Nausea	41 (61.1)	2 (2.9)	15 (60.0)	1 (4.0)	23 (74.1)	1 (3.2)	56 (60.9)	3 (3.2)
Vomiting	32 (47.7)	3 (4.4)	12 (48.0)	2 (8.0)	14 (45.1)	1 (3.2)	44 (47.8)	5 (5.4)
Fatigue	27 (40.2)	3 (4.4)	17 (68.0)	0 (0.0)	13 (41.9)	0 (0.0)	44 (47.8)	3 (3.2)
Anaemia	27 (40.2)	7 (10.4)	14 (56.0)	2 (8.0)	15 (48.3)	5 (16.1)	41 (44.6)	9 (9.8)
Alopecia	31 (46.2)	0 (0.0)	9 (36.0)	0 (0.0)	16 (51.6)	0 (0.0)	40 (43.5)	0 (0.0)
Decreased appetite	18 (26.8)	0 (0.0)	13 (52.0)	0 (0.0)	7 (22.5)	0 (0.0)	31 (33.6)	0 (0.0)
Constipation	18 (26.8)	1 (1.4)	10 (40.0)	1 (4.0)	9 (29.0)	0 (0.0)	28 (30.4)	1 (1.0)

*5-FU* 5-fluorouracil, *FOLFIRI* 5-fluorouracil plus folinic acid plus irinotecan, *RP2D* recommended phase 2 dose

^a^Patients in part 2 did not tolerate the 5-FU bolus as part of the FOLFIRI regimen. Therefore, dose escalation was discontinued and, although patients could continue veliparib, they were considered not evaluable. Data from the eight patients in part 2 were combined with part 3

^b^Includes 20 patients from part 1 and 11 patients from part 3

### Pharmacokinetics

**Fig. 2:**
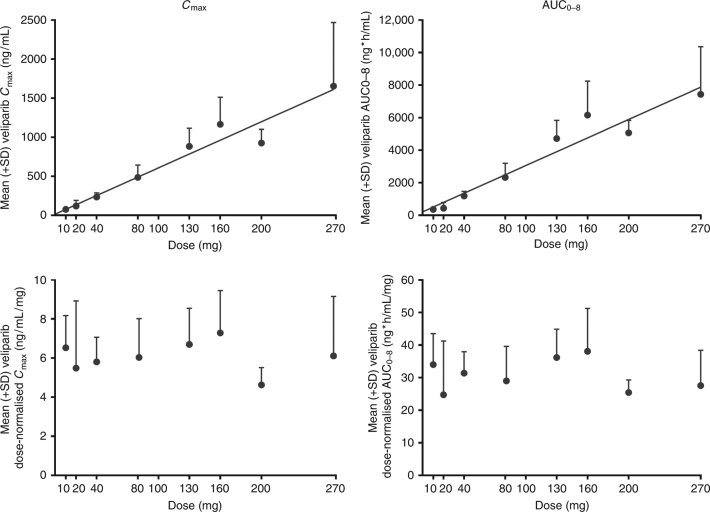
Evaluation of veliparib dose proportionality in part 1 of the study: mean (+SD) veliparib *C*_max_ and AUC (top) and dose-normalized *C*_max_ and AUC (bottom) presented vs increasing doses of veliparib (measured after the first administered dose of veliparib). Numbers of patients for each veliparib BID dose presented in the figure: *n* = 10 for 10 mg; *n* = 6 for 100 mg; *n* = 5 for 20 mg, 40 mg, and 200 mg; *n* = 4 for 80 mg and 130 mg; *n* = 7 for 160 mg; *n* = 9 for 270 mg *C*_max_, and *n* = 8 for 270 mg AUC_0-8_. AUC_0–8_ area under the plasma or serum concentration-time curve from time zero to hour 8, BID twice daily, *C*_max_ maximum plasma concentration, SD standard deviation

Veliparib exposure was approximately dose-proportional when co-administered with the study-specified FOLFIRI regimens (Fig. 2). The 90% CIs for the relative bioavailability (*C*_max_ and AUC from time zero to the time of last measurable concentration [AUC_*t*_]) of irinotecan, SN-38, dextroleucovorin ([R]-leucovorin), and levoleucovorin ([S]-leucovorin) in the presence (cycle 1 day 15) or absence (cycle 1 day 1) of veliparib co-administration were within the pre-established boundaries of 0.80 to 1.25 (Supplementary Table S2). This indicates that veliparib had no significant effect on the PK profiles of irinotecan or folinic acid. The median ratios of 5-FU concentrations at 2 h and 24 h after the start of infusion on cycle 1 day 15 to those on cycle 1 day 1 were 1.03 and 0.95, respectively. These results indicate that 5-FU concentrations were comparable, irrespective of veliparib co-administration.

### Exploratory efficacy findings

**Fig. 3:**
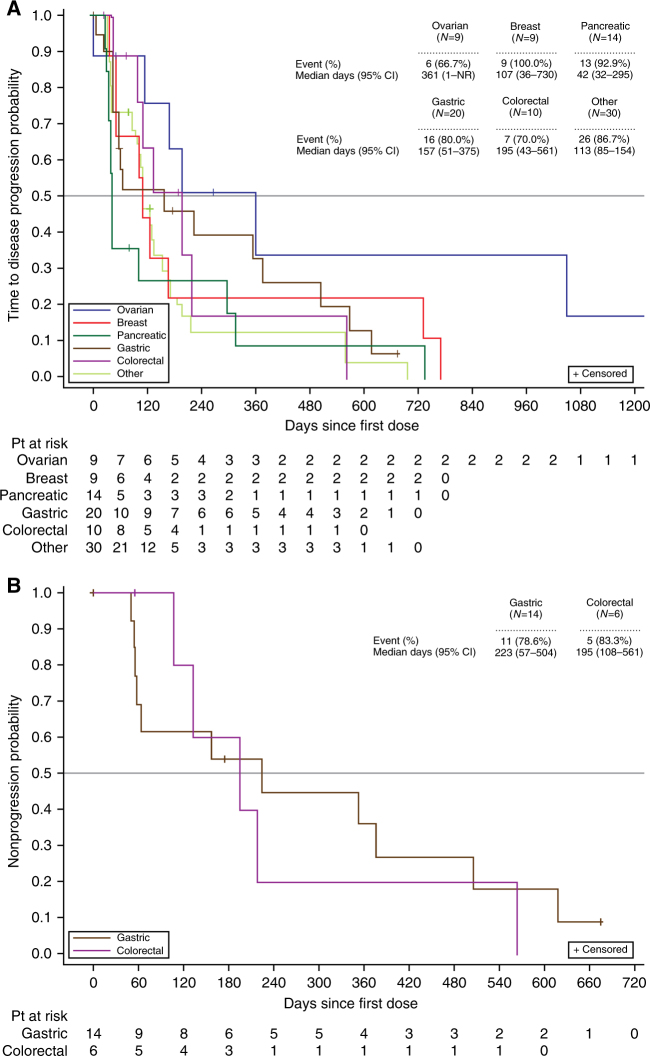
Kaplan–Meier curves for time to disease progression in **a**, all dosed patients, and **b**, expanded safety cohort. The number and percentage of patients with disease progression and the median number of days (and 95% CIs) to disease progression are detailed per cancer type. Censored events are depicted within each graph, and the numbers of patients at risk per cancer type are listed for each depicted timepoint. CI confidence interval, NR not reached

CR was not achieved by any patients. In total, there were 85 patients who had one or more measurable lesions at baseline. In these patients, the ORR was 17.6%. Patients with measurable disease ovarian cancer (*n* = 7) had the highest ORR (42.9%). ORRs by other indications are summarised in Table 3. Responder patients received a median of two prior oncology regimens (range: 0–9). The probability of remaining progression-free for 6 months (6-month TTP rate) varied across cancer types: 65% for patients with ovarian cancer (*n* = 9), 50.8% for patients with colorectal cancer (*n* = 10), 45.9% for patients with gastric cancer (*n* = 20), 26.8% for patients with pancreatic cancer (*n* = 14), 22.2% for patients with breast cancer (*n* = 9), and 21.2% for patients with other cancers (*n* = 30). The median TTP was longest in patients with ovarian cancer, at 361 days (range: 1–not reached) (Fig. 3a). For patients with colorectal or gastric cancer, the median TTP was 195 (range: 43–561) and 157 days (range: 51–375), respectively. The expanded safety cohorts included 14 patients with gastric cancer and six patients with colorectal cancer. In these two cohorts, the 6-month TTP rates were 53.8% and 60.0%, respectively. The gastric cancer safety expansion cohort (*n* = 14) had a median TTP of 223 days (range: 57–504); the colorectal cancer safety expansion cohort (*n* = 6) had a median TTP of 195 days (range: 108–561) (Fig. 3b). The median DOR was 560 days for patients who received irinotecan 150 mg/m^2^. For patients who received irinotecan 180 mg/m^2^, the median DOR was not determined due to small sample size (*n* = 2).

**Table 3:** Summary of objective response rates by tumour type

	Breast *n* = 8	Colorectal *n* = 9	Gastric *n* = 20	Ovarian *n* = 7	Pancreatic *n* = 14	Other *n* = 27	Overall *n* = 85^a^
ORR (CR + PR), *n* (%) (95% CI)	2 (25.0) (3.2–65.1)	2 (22.2) (2.8–60.0)	3 (15.0) (3.2–37.9)	3 (42.9) (9.9–81.6)	2 (14.3) (1.8–42.8)	3 (11.1) (2.4–29.2)	15 (17.6) (10.2–27.4)

*CI* confidence interval, *CR* complete response, *ORR* objective response rate, *PR* partial response

^a^Only patients with one or more measurable lesions at baseline were included in the analysis

## Discussion

PARP inhibition capitalises on the increased expression of PARP-1 and PARP-2 in a variety of tumours. Tumour cell reliance on PARP-mediated DNA repair provides the rationale for development of PARP inhibitors both as monotherapy and in combination with DNA-damaging chemotherapy and radiation therapy.^24,27^

Ovarian cancer is currently the only disease for which PARP inhibitors have received regulatory approval in Europe and the United States. The recent ARIEL2 phase 2 study, performed in patients with *BRCA* wild-type or *BRCA*-mutated ovarian carcinomas and genomic loss of heterozygosity (LOH), reported a longer progression-free survival (PFS) in patients with LOH-high platinum-sensitive ovarian carcinomas vs patients with LOH-low *BRCA* wild-type carcinomas.^28^ These results were confirmed in the phase 3 ARIEL3 study in patients with ovarian carcinoma, recurrent after response to platinum therapy.^29^ In the SOLO2 phase 3 study, maintenance treatment with olaparib in patients with platinum-sensitive relapsed ovarian cancer and a BRCA1/2 mutation led to significant PFS improvement vs placebo (median PFS: 19.1 vs 5.5 months).^30^ These results were consistent with those of previous phase 2 trials reporting clinical benefit for patients treated with olaparib, alone or in combination with chemotherapy.^31,32^ Similar results were recently described for the ENGOT-OV16/NOVA phase 3 trial with the PARP-1 and PARP-2 inhibitor niraparib, administered in patients with platinum-sensitive, recurrent ovarian cancer.^33^

To date, the novel PARP-1 and -2 inhibitor veliparib has been evaluated in phase 1 and 2 trials as monotherapy^34,35^ and in combination with multiple chemotherapy regimens, including temozolomide,^36,37^ topotecan,^38^ and carboplatin/paclitaxel.^39^ Recent data from a phase 1 study of veliparib in combination with irinotecan in patients with advanced solid tumours demonstrated that veliparib at 40 mg BID on days 1–14 of a 21-day cycle was well tolerated in combination with irinotecan (100 mg/m^2^; day 1 and 8), with preliminary evidence of antitumour activity and reductions in PARP levels in paired tumour biopsies.^40^ Additional evidence of the tolerability of veliparib in combination with irinotecan has been reported in patients with triple-negative breast cancer. The cohort of patients with germline *BRCA* mutation had a preliminary response rate of 88%, although the number of patients in this cohort was limited.^41^

This phase 1 study identified the RP2D of veliparib in patients with advanced solid tumours as 200 mg BID with bimonthly FOLFIRI on the basis of an irinotecan dose of 150 mg/m^2^ or 180 mg/m^2^ without 5-FU bolus. Veliparib in combination with FOLFIRI was generally well tolerated, and the MTD was not reached, on the basis of the Bayesian CRM guiding dose escalation. Limitations of the CRM include the pre-specification of prior toxicity probabilities, which may be distorted and result in the need to adjust the model, as was the case in this study, from the initially projected upper dose range of veliparib 80 mg. The type and incidence of TEAEs were similar between patients who received irinotecan 150 mg/m^2^ or 180 mg/m^2^, and were consistent with the known side effects of FOLFIRI, which include grade 3/4 neutropenia and gastrointestinal symptoms.^42^ The favorable tolerability profile of the regimen was also supported by data on duration of therapy. It is probable that our strategy of utilising veliparib intermittently, only when FOLFIRI was being dosed, improved the ability to achieve the target dose for veliparib at a dose level that brings about adequate PARP inhibition. Additionally, it appeared that avoiding the bolus dosing of 5-FU improved tolerance of this regimen. Nonetheless, few patients discontinued therapy due to a TEAE, either with or without the bolus 5-FU in this trial. Veliparib achieved approximate dose proportionality and showed no effect on the PK of any FOLFIRI component. There was preliminary evidence of antitumour activity, with the combination of veliparib and FOLFIRI yielding an ORR of 17.6%; patients with ovarian and breast cancer experienced the greatest antitumour activity, with an ORR of 42.9% and 25.0%, respectively. An ORR similar to that of patients with breast cancer was also observed in patients with colorectal cancer (ORR = 22.2%). Patients with pancreatic cancer had an ORR of 14.3%. The long median DOR in patients who received irinotecan 150 mg/m^2^ also attests to the antitumour activity and tolerability of the combination therapy.

The precise mechanism of action of PARP inhibitors is an ongoing area of research, and preclinical data suggest that their activity may depend on different mechanisms of action. One possible theory for the mechanism of action of PARP inhibitors in tumours with HRR deficiencies is synthetic lethality due to the simultaneous blockade of the pathways involved in base excision repair and HRR DNA repair.^43,44^ An additional proposed mechanism of action is PARP trapping, whereby PARP inhibitors act as DNA poisons by trapping PARP on damaged DNA, resulting in cytotoxic PARP–DNA complexes. Interestingly, this trapping mechanism seems to occur at exposures that are higher than those required for catalytic inhibition.^45^ *BRCA*-mutated tumour cells appear to be especially sensitive to PARP inhibition by synthetic lethality.^46^ In this study, *BRCA* mutation leading to impaired HRR may have resulted in improved response rates in patients with ovarian and breast cancer. In other tumours, the heavy prior exposure to cytotoxic chemotherapy and radiation therapy could have negatively influenced the effect of veliparib plus FOLFIRI. This has implications not only in terms of selection of patients for veliparib treatment, but also for proper sequencing of veliparib, which might yield optimal outcomes when used before platinum-based chemotherapy or as maintenance therapy for high-risk, genetically susceptible patients following standard treatment. However, this study has as a limitation the fact that no formal genetic testing was performed (e.g., *BRCA* mutations).

In this trial, the use of veliparib prior to and during chemotherapy was designed to maximise the potential synergy between PARP inhibition and DNA-damaging agents, particularly irinotecan. While FOLFIRI is an active regimen, the results demonstrated broad activity, including significant stable disease and response rates of over 15% in a previously treated patient population. These results are encouraging and indicate that this strategy is appropriate to maximise the benefit and tolerance of the addition of a PARP inhibitor to chemotherapy.

In conclusion, this phase 1 dose-escalation study demonstrates that veliparib and FOLFIRI can be safely combined in patients with advanced solid tumours. Preliminary data show antitumour activity in several tumour types. The findings reported herein support further evaluation of veliparib in combination with FOLFIRI. Studies have been initiated in first-line colorectal cancer and in second-line pancreatic cancer to further assess this regimen.

## Electronic supplementary material

Supplementary materials
