# Supplementary material for: A phase 1 dose-escalation study of veliparib with bimonthly FOLFIRI in patients with advanced solid tumours
Source: Br J Cancer. 2018 Mar 12;118(7):938–46. doi: 10.1038/s41416-018-0003-3 (PMC5933261; doi:10.1038/s41416-018-0003-3)
Supplement: Supplementary file 1 — Supplementary materials [file 41416_2018_3_MOESM1_ESM.docx]

| **Supplementary Table S1: Treatment regimens for parts 1, 2, and 3 of the study** | | |
| --- | --- | --- |
| **Dose level** | **Veliparib^a^** | **FOLFIRI^b^**  **(Irinotecan/folinic acid [leucovorin^d^]/5-FU^e^)** |
| **Part 1 (n=67)** | | |
| 1 (n=10^c^) | 20 mg/day (10 mg BID) | 150/400/2,400 mg/m^2^ |
| 2 (n=5) | 40 mg/day (20 mg BID) |  |
| 3 (n=5) | 80 mg/day (40 mg BID) |  |
| 4 (n=4) | 160 mg/day (80 mg BID) |  |
| 5 (n=4) | 260 mg/day (130 mg BID) |  |
| 6 (n=9) | 320 mg/day (160 mg BID) |  |
| 7 (n=20) | 400 mg/day (200 mg/BID) |  |
| 8 (n=10) | 540 mg/day (270 mg/BID) |  |
| **Part 2 (n=8)^f^** | | |
| 1 (n=8) | 200 mg/day (100 mg BID) | 180/400/400–2,400 mg/m^2^ |
| **Part 3 (n=17)** | | |
| 1 (n=6) | 200 mg/day (100 mg BID) | 180/2,400 mg/m^2^ |
| 2 (n=11) | 400 mg/day (200 mg BID) |  |
| Abbreviations: 5-FU, 5-fluorouracil; BID, twice daily; FOLFIRI, 5-fluorouracil plus folinic acid plus irinotecan; MTD, maximum tolerated dose.  ^a^In part 1 of the study, veliparib was administered on days 15–19 of cycle 1, and on days 1–5 and 15–19 of each subsequent 28-day cycle. In parts 2 and 3, veliparib was administered on day 15 of cycle 1, and on days 1–5 and 15–19 of each subsequent 28-day cycle.  ^b^In all three parts of the study, the FOLFIRI regimen was administered on days 1–3 and 15–17 of each 28-day cycle.  ^c^Four patients were also dosed with bolus 5-FU 400 mg/m^2^ as part of the original FOLFIRI regimen.  ^d^If leucovorin was not available due to shortages, levoleucovorin may have been substituted for leucovorin. The dose of levoleucovorin is 50% of the usual dose of leucovorin; thus, the dose was adjusted to 200 mg/m^2^ of levoleucovorin.  ^e^If, in dose cohort 1, fewer than four of six patients were evaluable to start cycle 2, the original FOLFIRI regimen was considered not appropriate to combine with veliparib due to poor tolerability and, thus, part 2 was to be terminated.  ^f^Patients were also dosed with bolus 5-FU 400 mg/m^2^. | | |

| **Supplementary Table S2: Relative bioavailability assessment for the effect of veliparib on irinotecan, SN-38, and folinic acid (leucovorin) pharmacokinetics** | | | | | | | | | | | |
| --- | --- | --- | --- | --- | --- | --- | --- | --- | --- | --- | --- |
| **Regimens Test vs reference** | **N** | **Pharmacokinetic parameters** |  |  | | | | **Relative bioavailability** | | | |
|  |  |  | **Central value** | | | | | **Point estimate** | | | **90% confidence interval** |
|  |  |  | **Test** | **Reference** | | | |  |  |  |  |
|  |  |  | **Irinotecan** | | | | | | | | |
| FOLFIRI + veliparib vs FOLFIRI alone | 59 | C_max_ (µg/mL) | 1.786 | 1.775 | | | | 1.006 | | | 0.962–1.053 |
|  | 59 | AUC_0–24_ (µg•h/mL) | 10.77 | 10.62 | | | | 1.014 | | | 0.964–1.067 |
|  |  |  | **SN-38** | | | | | | | | |
| FOLFIRI + veliparib vs FOLFIRI alone | 59 | C_max_ (µg/mL) | 0.01765 | | 0.01831 | | 0.964 | | | 0.907–1.025 | |
|  | 59 | AUC_t_ (µg•h/mL) | 0.1578 | | 0.1579 | | 1.000 | | | 0.904–1.105 | |
|  |  |  | **(R)-Leucovorin** | | | | | | | | |
| FOLFIRI + veliparib vs FOLFIRI alone | 49 | C_max_ (µg/mL) | 39.86 | | 41.87 | | 0.952 | | | 0.910–0.996 | |
|  | 49 | AUC_t_ (µg•h/mL) | 172.2 | | 180.4 | | 0.955 | | | 0.915–0.997 | |
|  |  |  | **(S)-Leucovorin** | | | | | | | | |
| FOLFIRI + veliparib vs FOLFIRI alone | 49 | C_max_ (µg/mL) | 13.27 | 14.22 | | 0.933 | | | 0.857–1.015 | | |
|  | 49 | AUC_t_ (µg•h/mL) | 28.47 | 29.36 | | 0.970 | | | 0.898–1.048 | | |
| Abbreviations: AUC_0–24_, area under the plasma or serum concentration-time curve from time zero to 24 hours; AUC_t_, area under the plasma or serum concentration-time curve from time zero to the time of last measurable concentration; C_max_, maximum plasma concentration; FOLFIRI, 5-fluorouracil plus folinic acid plus irinotecan.  Note: FOLFIRI administered with veliparib on cycle 1 day 15 (test); FOLFIRI administered alone on cycle 1 day 1 (reference). | | | | | | | | | | | |
